# Pulmonary Lobectomy for Chronic Pulmonary Vein Occlusion after Catheter Ablation for Atrial Fibrillation: A Case Report and Literature Review

**DOI:** 10.70352/scrj.cr.24-0034

**Published:** 2025-02-08

**Authors:** Satoshi Suzuki, Nobuhiro Izumi, Kazuya Kishimoto, Hirotaka Kinoshita, Takuya Tanimura, Kantaro Hara, Hidetoshi Inoue, Takuma Tsukioka, Junichi Soh

**Affiliations:** Department of Thoracic Surgery, Osaka Metropolitan University Graduate School of Medicine, Osaka, Osaka, Japan

**Keywords:** atrial fibrillation, ablation, pulmonary vein stenosis, pulmonary necrosis, pulmonary lobectomy

## Abstract

**INTRODUCTION:**

Pulmonary vein stenosis (PVS) is known as one of the chronic complications after catheter ablation for atrial fibrillation (AF). The endovascular approach is a less invasive treatment option for PVS, while pulmonary lobectomy is also chosen, especially for patients with pulmonary vein occlusion. Here, we present a case of pulmonary vein occlusion accompanied by pulmonary necrosis that was successfully treated by pulmonary lobectomy.

**CASE PRESENTATION:**

A 65-year-old man underwent catheter ablation for AF along with administration of anticoagulants at his previous hospital. After treatment for 6 months, hemoptysis appeared, and chest computed tomography (CT) showed an infiltration shadow in the lower lobe of the left lung. The patient was admitted to the hospital, and antibiotic therapy was initiated. Despite 10 days of antibiotic therapy, there was no improvement, and the lung infiltration worsened. Therefore, on the 10th day of hospitalization, the patient was transferred to our institute. A bloody lavage fluid was obtained under a bronchoalveolar lavage, suggesting alveolar hemorrhage. Then, a contrast-enhanced chest CT scan confirmed a complete occlusion of the left inferior pulmonary vein with suspicion of pulmonary necrosis. We performed a left lower lobectomy under a video-assisted thoracic approach. The lower lobe of the left lung was dark red with a bad smell, and there was 500-ml bloody pleural fluid. Intraoperative transesophageal echocardiography showed no thrombus in the inferior pulmonary vein. The surrounding tissue of the occlusion area of pulmonary vein was sclerotic and inflammatory with firm adhesions to the vagus nerve. The inferior pulmonary vein was separated on the non-hardening peripheral side of the occlusion point using a stapler. Pathological examination confirmed multiple hemorrhagic infarctions in the parenchyma. The patient was discharged on the 8th postoperative day, and there was no recurrence of hemoptysis at 6 months postoperatively.

**CONCLUSIONS:**

We successfully treated patients with pulmonary vein occlusion following catheter ablation through pulmonary lobectomy. While endovascular treatment is less invasive and remains the first choice for PVS, lobectomy should be considered in patients with complete occlusion, especially when accompanied by pulmonary necrosis, or in recurrent patients after endovascular treatment.

## Abbreviations

CT
: computed tomography
PVS
: pulmonary vein stenosis
AF
: atrial fibrillation

## INTRODUCTION

The number of catheter ablation for atrial fibrillation (AF) is increasing, and pulmonary vein stenosis (PVS) has been reported as one of the complications.^1)^ Balloon dilation, stenting, and pulmonary vein angioplasty have been reported as treatment options, but there have also been reports of pulmonary resection for severe stenosis. We herein describe a case of complete occlusion of the left inferior pulmonary vein accompanied by pulmonary necrosis after catheter ablation that required left inferior lobectomy.

## CASE PRESENTATION

**Fig. 1:**
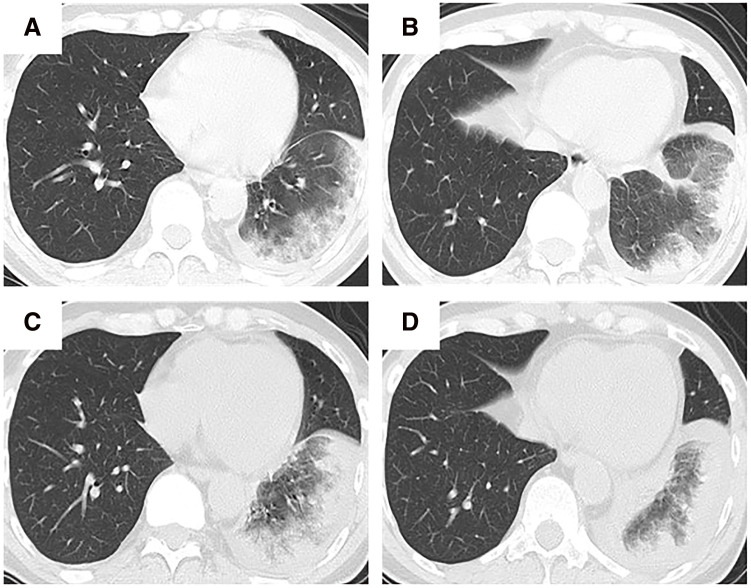
Clinical course of lung infiltration after catheter ablation. (**A** and **B**) Chest computed tomography (CT) scans 6 months after catheter ablation just before the beginning of antibiotic therapy. (**C** and **D**) Chest CT scans 10 days after antibiotic therapy.

**Fig. 2:**
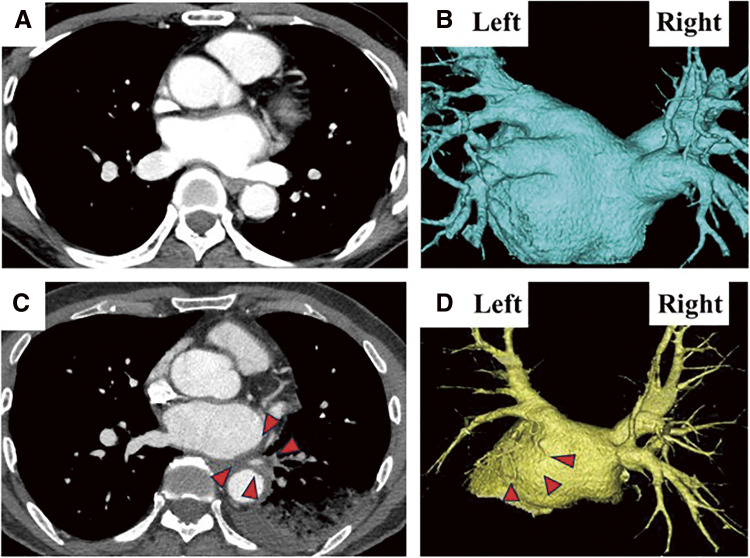
Pulmonary vein occlusion occurred after catheter ablation. (**A** and **B**) 2-dementional (2-D) axial images and 3-D construction images of contrast-enhanced chest CT scan before catheter ablation. (**C** and **D**) 2-D axial images and 3-D construction images of contrast-enhanced chest CT scan 6 months after catheter ablation.

**Fig. 3:**
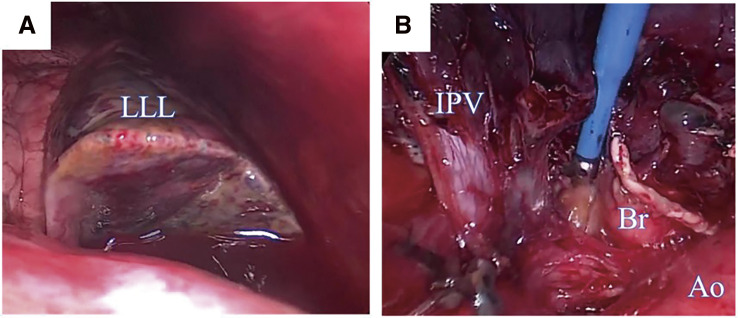
Intraoperative images. (**A**) The swollen left lower lobe (LLL) with a dark reddish visceral pleura and a malodorous pleural effusion. (**B**) The left inferior pulmonary vein and LLL bronchus stump and descending aorta. IPV, inferior pulmonary vein; Br, LLL bronchus stump; Ao, descending aorta

**Fig. 4:**
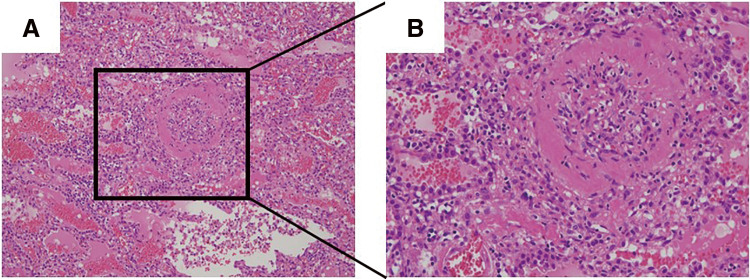
Representative histological images of the resected lung.

A 65-year-old man underwent catheter ablation for AF along with administration of anticoagulants at his previous hospital. After 6 months of treatment, hemoptysis appeared and chest computed tomography (CT) showed an infiltration shadow in the lower lobe of the left lung (**Figs. 1A** and **1B**), and he was diagnosed as bacterial pneumonia. The patient was admitted to the hospital, anticoagulation was stopped, and antibiotic therapy was initiated. Despite 10 days of antibiotic therapy, there was no improvement, and the infiltration shadow worsened (**Figs. 1C** and **1D**). Therefore, on the 10th day of hospitalization, the patient was transferred to our institute. A bronchoalveolar lavage, which was performed under suspicion of organizing pneumonia, revealed a bloody lavage fluid, suggesting an alveolar hemorrhage. A PVS after catheter ablation was considered and a contrast-enhanced chest CT scan confirmed a complete occlusion of the left inferior pulmonary vein (**Fig. 2**). In addition, pulmonary necrosis was suspected because an infiltrating shadow just below the pleura and a large amount of pleural effusion was observed. We performed a left lower lobectomy under a video-assisted thoracic approach. The lower lobe of the left lung was dark red with a bad smell, and there was 500 ml of bloody pleural fluid (**Fig. 3A**). Transesophageal echocardiography showed no thrombus in the inferior pulmonary vein, but surrounding tissue of the occlusion area of pulmonary vein was sclerotic and inflammatory with firm adhesions to the vagus nerve. After the bronchus was transected, the surrounding tissue around the inferior pulmonary vein was dissected and palpation confirmed that the peripheral side of the occlusion area of the pulmonary vein was not sclerosed (**Fig. 3B**). So, the inferior pulmonary vein was separated on the non-hardening peripheral side of the occlusion using Endo GIA Ultra Universal Stapler Tri-Staple 2.0 Camel (Covidien Japan Inc., Tokyo, Japan). Pathological examination revealed that PVS with thickened pulmonary vein walls in all layers and multiple hemorrhagic infarctions in the lung parenchyma were observed (**Fig. 4**). The patient was discharged on the eighth postoperative day, and there was no recurrence of hemoptysis at 6 months postoperatively.

## DISCUSSION

Catheter ablation for AF is a well-established therapy for rhythm control and reported that catheter ablation may have superior efficacy to anti-arrhythmic drugs as an initial rhythm control strategy in patients with symptomatic AF.^2)^ PVS is one of the complications of catheter ablation for AF. The frequency of PVS has been estimated to range from 0.7% to 3.4%,^3,4)^ and previous studies reported that 0.29% of patients who underwent catheter ablation for AF require invasive treatment due to PVS.^5)^ PVS is reported to be more common on the left side, which is related to the anatomy, such as the small diameter of the left inferior PV, the relatively cranial orientation of the left superior vein, or the position of Coumadin ridge near the left atrial appendage forcing ablation closer to the venous ostium.^6)^

Symptoms of PVS vary from hemoptysis, dyspnea, coughing, and chest pain, and there have been reports of asymptomatic cases.^4)^ It is difficult to diagnose PVS because symptoms are non-specific.^7)^

PVS can cause pulmonary infarction in severe cases, and the most common appearance of pulmonary infarction is reported to be a infiltrative shadows just below the pleura in CT scan as shown in our case.^8)^ However, our patient was initially diagnosed with bacterial pneumonia, and it took approximately two weeks to diagnose PVS. Qureshi et al. reported that the condition was initially misdiagnosed in 17 of 19 patients after the onset of symptoms from PVS with a delay to the correct diagnosis of 16 weeks.^9)^ For accurate diagnosis, it is important to always consider the possibility of this disease when infiltrative shadows just below the pleura are observed in CT scan of patients who have undergone catheter ablation for AF.

Although PVS is often treated with endovascular treatment such as percutaneous balloon dilation and stenting, these procedures are associated with restenosis rates of 49% and 25%, respectively, during a follow-up period of 36 months.^6)^ Surgical treatment is indicated for younger patients or occlusion cases because long-term antithrombotic therapy is necessary and endovascular treatment is difficult in cases of pulmonary vein occlusion.^10,11)^ Since our case indicated that the pulmonary vein was completely occluded and pulmonary necrosis due to pulmonary infarction was highly suspected, we decided on a surgical resection and multiple hemorrhagic infarcts were histologically confirmed. To the best of our knowledge, no previous reports have provided detailed descriptions of intraoperative findings or surgical techniques for lobectomy in cases of PVS. We believe that the approach to separating the pulmonary vein in PVS is a critical consideration for surgeons. If a thrombus is present in the pulmonary vein, it is crucial to remove it completely before separating the vein to reduce the risk of further thrombosis and subsequent complications, including cerebral infarction. Conversely, if no thrombus is detected in pulmonary vein, separating the pulmonary vein on the peripheral side of the stenosis may be an acceptable surgical strategy. In our case, the absence of thrombus in the inferior pulmonary vein was confirmed using transesophageal echocardiography, and the occlusion point was identified by palpating the sclerosed lesion. We then separated the pulmonary vein using a stapler on the non-hardening peripheral side of the occlusion to preserve the vagus nerve, achieving favorable outcomes.

There have been reports of cases requiring lobectomy, and a search on PubMed for “ablation”, “pulmonary vein stenosis”, and “lobectomy“ revealed 11 case reports (**Table 1**).^11–21)^ The median age was 51 years, and 9 patients (82%) were male. The median time from catheter ablation to symptom appearance was 6 months, and 9 patients (82%) had complete occlusion of pulmonary vein. Ten patients (91%) were left lobe, and lobectomy was selected without endovascular treatment in 5 patients (45%). The postoperative course was noted in 7 of the 11 patients (64%), the median postoperative observation period was 18 months (1–33 months), and none of the patients had a recurrence of PVS. Lobectomy is a highly curative treatment for PVS and should be considered in cases of complete occlusion or restenosis after endovascular treatment.

**Table 1:** Summary of 11 patients who underwent lobectomy for pulmonary vein stenosis after ablation

Author (year)	Age	Sex	Chief complaint	Time to onset (month)	Degree of stenosis	Resection lobe	Reasons for lobectomy	Follow-up (month)
Yang^21)^ (2007)	55	M	Dyspnea	1.5	Occlusion	LLL	Recurrence	10
Steliga^20)^ (2010)	51	F	Chest pain	6	Occlusion	LUL	Occlusion	1
Libretti^19)^ (2012)	17	M	Dyspnea	Unknown	Stenosis	LLL	Recurrence	33
Lo^11)^ (2016)	47	F	Hemoptysis	6	Occlusion	RLL	Occlusion	18
Papakonstantinou^17)^ (2018)	50	M	Hemoptysis	8	Occlusion	LUL	Occlusion	Unknown
O’Gorman^18)^ (2019)	51	M	Dyspnea	4	Occlusion	LUL	Recurrence	Unknown
Yu^16)^ (2019)	62	M	Hemoptysis	5	Occlusion	LUL	Occlusion	30
Xuan^15)^ (2020)	38	M	Hemoptysis	0.3	Occlusion	LUL	Recurrence	Unknown
Matsumoto^14)^ (2021)	48	M	Hemoptysis	36	Stenosis	LLL	Recurrence	12
Akiki^13)^ (2022)	73	M	Hemoptysis	24	Occlusion	LLL	Recurrence	18
Murray^12)^ (2022)	68	M	Dyspnea	12	Occlusion	LLL	Occlusion	Unknown

M, male; F, female; RLL, right lower lobe; LUL, left upper lobe; LLL, left lower lobe; Recurrence, recurrence after endovascular treatment.

## CONCLUSIONS

We presented a case who underwent pulmonary lobectomy for pulmonary vein occlusion after catheter ablation for AF. While endovascular treatment is less invasive and remains the first choice for PVS, lobectomy should be considered in complete occlusion cases, especially when accompanied by pulmonary necrosis, or in recurrent cases after endovascular treatment.

## DECLARATIONS

### Funding

Not particular.

### Authors' contributions

SS and JS wrote the manuscript.

SS, NI, KK, HK, TTa, KH, HI, and TTsu collected the patient’s data.

All authors read the manuscript and approved the paper.

### Availability of data and materials

Not applicable.

### Ethics approval and consent to participate

This study was approved by the Osaka Metropolitan University Ethic Committee (reference number 2019-006). Informed consent was obtained from patient in written form.

### Consent for publication

Written informed consent was obtained from the patient for publication of this case report and accompanying images.

### Competing interests

There are no financial and non-financial competing interests for all authors.
